# Lambs Grazing With Adult Ewes Prefer Forbs With High‐Nutrient Content in Native Grasslands Dominated by *Leymus chinensis* and *Stipa grandis*

**DOI:** 10.1002/ece3.70609

**Published:** 2024-11-18

**Authors:** Pengzhen Li, Zhenhao Zhang, Thomas A. Monaco, Yuping Rong

**Affiliations:** ^1^ Department of Grassland Resource and Ecology, College of Grassland Science and Technology China Agricultural University Beijing China; ^2^ U.S. Department of Agriculture, Agricultural Research Service, Forage and Range Research Laboratory Utah State University Logan Utah USA

**Keywords:** dietary composition, ewe, foraging behavior, lamb, vegetation diversity

## Abstract

Grazing livestock in grasslands face the challenge of obtaining sufficient nutrition due to uneven distribution of plant species and fluctuating vegetation productivity and nutrient levels. In northern China, *Leymus chinensis* and *Stipa grandis* are the dominant perennial species in native grasslands, but they provide limited nutrition compared to forbs with higher crude protein (CP) content. While dietary ingredients can affect the nutritional intake of grazing livestock, the influence of different grazing strategies on dietary selection remains unclear. In this study, three sheep grazing strategies (lambs alone, mixed lambs and ewes, and ewes alone) were used to explore dietary selection. We investigated the influence of vegetation characteristic (above‐ground biomass production, height, and species diversity) and foraging behavior (feed intake, organic matter digestibility, and daily grazing time) on the mechanisms of dietary selection (taxonomic family richness and composition). Forage consumption across the grazing strategies revealed that species from Poaceae, Rosaceae, and Cyperaceae families were frequently consumed. Both ewes and lambs in the mixed‐grazing strategy preferentially consumed forbs with diverse species composition (Jacob's *D* > 0), which contained higher CP than those available in the overall vegetation (*p* < 0.05). In addition, dietary richness was significantly (*p* < 0.05) influenced by vegetation species diversity except for animals in the lambs alone strategy. Compared to lambs in mixed‐grazing, lambs alone had both greater daily grazing time and consumption of grass with lower digestibility (*p* < 0.05). Our study is the first to demonstrate that lambs can develop a similar dietary selection and behavioral pattern when grazing with adult ewes in temperate grasslands. Our study indicates that the conservation of species diversity in native grasslands is critically beneficial to livestock nutrition.

## Graphical abstract

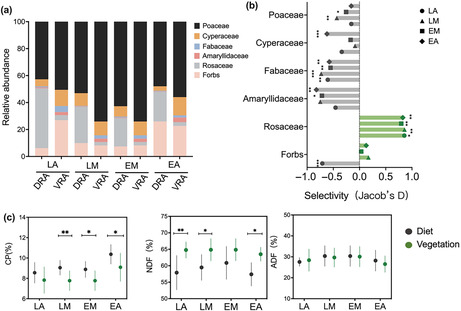

## 1 Introduction

A key issue for livestock production in native grassland is discord between vegetation production and dietary requirements for animal production. Grazing animals selectively forage from available feed resources in grasslands (Guo et al. 2021) and adjust their consumption to intake higher nutrient plant species (Spitzer et al. 2020). This occurs because temperate grasslands are mainly composed of grasses with low nutrient content (Oñatibia, Aguiar, and Oesterheld 2023; Ren et al. 2018; Zhang et al. 2022), wherein large herbivores require a greater abundance of nutrient‐rich plants for their performance (Stephens and Krebs 1987). Given the highly variable nature of species composition and vegetation abundance in grasslands, the mechanisms responsible for variation in dietary selection among foraging strategies of grazing herbivores in native grasslands remain unclear.

Grazing animals selectively consume plant species with higher nutrient content, which is believed to increase protein levels and decrease fiber and lignin concentrations of their diet (Mellado et al. 2011). This selective behavior can enhance digestibility and nutritional intake to meet their specific nutrient requirements (Pain et al. 2015). However, high spatial and temporal variability both in the quantity and quality of vegetation in native grasslands can strongly influence dietary selection of grazing animals (Walker et al. 2023). In addition, vegetation production changes with extreme climatic events, which might compel animals to continuously regulate their foraging strategies to secure sufficient resources (Oñatibia, Aguiar, and Oesterheld 2023; Vose et al. 2016). For example, grazing animals vary their seasonal dietary selection to enhance their utilization of nitrogen and energy‐rich forage (Guo et al. 2021). Furthermore, the dietary preference of goats shifts from herbaceous annual plants to woody shrubs under continuous drought conditions (Schroeder et al. 2019). These studies indicate that environmental conditions and foraging behavior are key factors influencing dietary preferences of grazing animals, yet our understanding of the interactions between these factors are limiting (Bolzan et al. 2020; Spitzer et al. 2023; Walker et al. 2023).

Animal developmental stage and sex, which directly influence nutritional requirements (Benvenutti, Gordon, and Poppi 2008) and detoxification abilities (Provenza et al. 2003), lead to distinct differences in nutrient acquisition and foraging behavior among herbivores (Benvenutti, Gordon, and Poppi 2008). In addition, because animals are born with inherent, physiology‐ and morphology‐related behavioral predispositions that influence their feeding motivations (Launchbaugh and Howery 2005), their food choices are ultimately formed depending on their environment and social groups (Provenza, Pfister, and Cheney 1992). For example, in different grazing strategies, animals often imitate or follow the forage strategies of other individuals to obtain maximum benefit (Villalba, Provenza, and Han 2004). These studies indicate that a greater understanding of the role of social relationships among grazing strategies and the mechanisms affecting foraging decisions merit greater attention among animals that span developmental stage.

In most management and production practices, lambs and ewes are separated due to targeted growth rates, market weights, and breeding schedules (Barkley 2014; Napolitano, De Rosa, and Sevi 2008; Urbano et al. 2017). However, this management strategy may expose lambs to base food choices, solely environmental factors, and food supply dynamics (Weary, Jasper, and Hötzel 2008). When lambs graze alone, it can lead to behavioral and physiological stress and affect their performance by reducing daily weight gain and carcass weight (Pascual‐Alonso et al. 2015; Pullin et al. 2017). For example, lambs grazing alone exhibited longer grazing time compared to mixed‐grazing with adult sheep in grassland pastures of southern Brazil (da Silva et al. 2020). When lambs are separated from their mother, they experience foraging stress caused by changes in pasture growth rates and changes in pasture quality and quantity due to factors such as erratic weather (Poli et al. 2020). However, no quantitative studies have assessed how different grazing strategies affect foraging behavior and dietary species selection.

Livestock production in native grassland systems can be unstable and fragile due to highly variable forage production and quality. Consequently, it is critical to understand livestock utilization of grasslands under various grazing strategies and identify which strategy may be most beneficial to simultaneously increase livestock performance and sustainably utilize forage resources. In this study, we established three grazing strategies, including lambs alone (LA), ewes alone (EA), and mixed lambs and ewes (LM/EM) to explore the following: (1) dietary variation and the associated behavioral patterns among animals in the different grazing strategies and (2) influence of plant community composition and nutritional status on animal foraging choices.

## 2 Materials and Methods

### 2.1 Site Description

**FIGURE 1:**
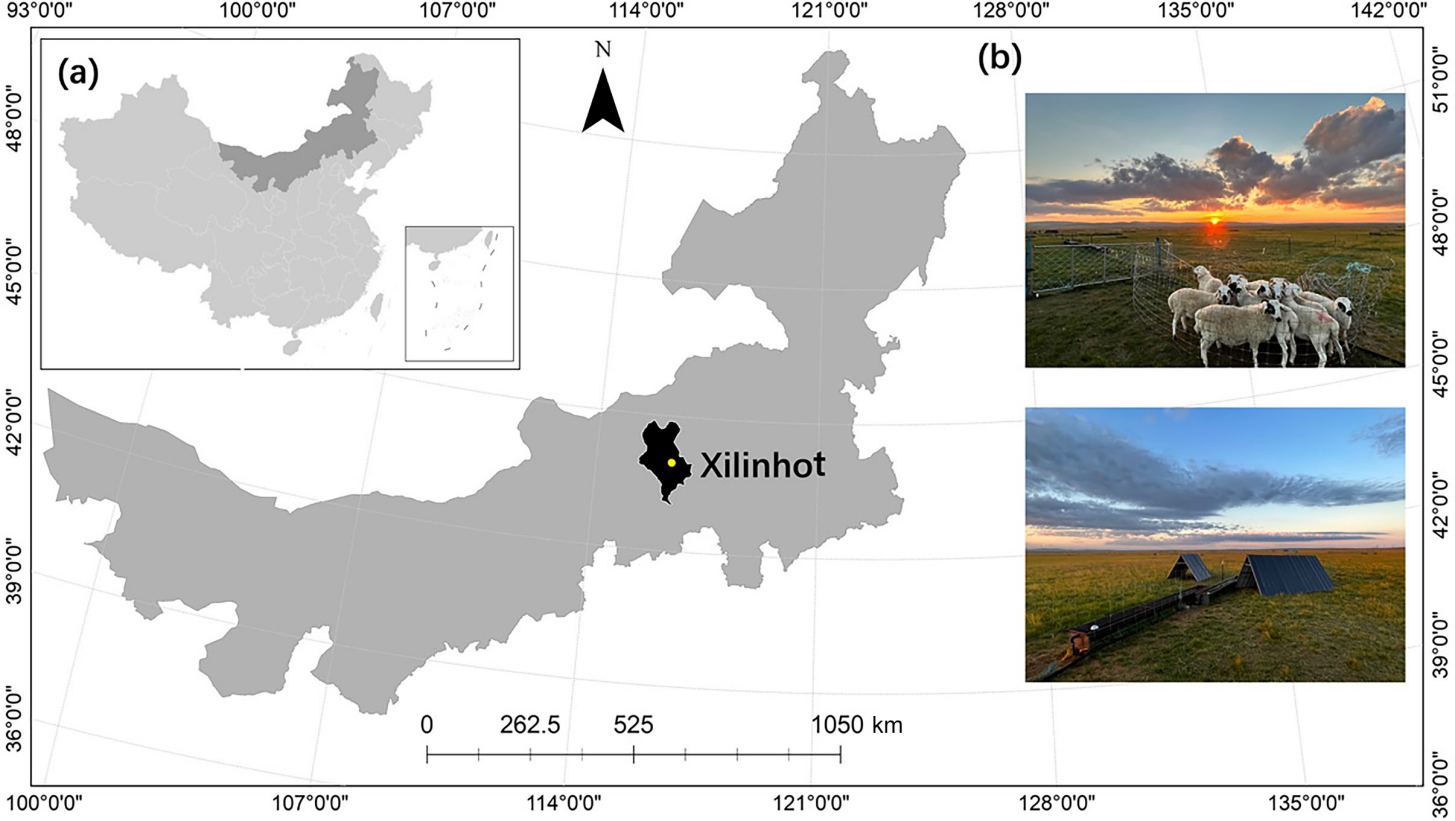
Location of the study site (a). Photographs from the experiment plots (b).

The study was conducted at the Grassland Ecosystem Research Station (44°00′ N, 116°26′ E, elevation 1150 m), a temperate grassland located in Xilinhot, Inner Mongolia, China (Figure 1). Temperatures vary from a mean winter (December–February) minimum of −16.5°C to a mean summer (June–August) maximum of 20.7°C. The mean annual precipitation is between 170 and 370 mm, about 80% of which falls in the growing season from May to August. Dominant species include the perennial grasses, *Leymus chinensis* and *Stipa grandis*, which collectively constitute about 50%–70% of the total above‐ground biomass. Other species include two grasses ( *Cleistogenes squarrosa* and *Agropyron cristatum* ), one sedge (*Carex korshinskyi*), and one perennial herb (*Sibbaldianthe bifurca*). The total cover of vegetation is 40%–50%, and the average height is 10–15 cm. The site has a dark chestnut soil (Calcic Chernozem according to ISSS Working Group RB, 1998), with a loamy‐sand texture (Li et al. 2019).

### 2.2 Grazing Experiment

Our study used three continuous grazing strategies that were originally established for a broader set of experiments with varying stocking rates and animal groups. The plot size of each grazing strategy is 2‐ha with three replicates. During the grazing period, the stocking rates for LA, LM/EM, and EA were 334 ± 28.2, 369 ± 18.8, and 347 ± 10.4 kg live animal weight per ha, respectively. Hereafter, grazing strategies were labeled as LA, LM/EM, and EA and included 10 male lambs, four adult ewe‐male lamb pairs (having mother–child relationships), and six adult ewes per plot, respectively. The study began in 2022, and all measurements were made during the growing season between July and September.

A total of 72 local‐breed Ujimqin sheep were used in this study, including 42 male lambs with an average weight of 26 ± 1.7 kg and 30 adult ewes with an average weight of 54 ± 1.7 kg. The lambs were about 3 months old, and adult ewes about 3–4 years old, not pregnant. After selection, they were orally administered an anthelmintic against internal and external parasites, ear‐tagged for identification, and then allocated randomly to experimental plots according to the grazing strategies. Sheep were allowed to graze throughout the day, and a shelter was established in each plot for rest. They had free access to a mineral block, salt, and water, and no supplement was given during grazing periods.

### 2.3 Vegetation Sample Collection and Nutritional Analyses

Vegetation samples were collected on 20 July, 15 August, and 10 September in 2022. Ten sampling quadrats (50 × 50 cm) were randomly placed within each of the three 2‐ha plots, resulting in a total of 30 quadrats used to evaluate above‐ground biomass, species diversity, and vegetation height for each grazing strategy. Green vegetation within quadrats was cut to ground level using scissors and identified to species level. In this area, 37 native plant species were identified, mainly from five plant functional groups: Grass, sedge, legume, forb, and shrub. In this experiment, combining both plant growth form and above‐ground biomass, species were categorized into Poaceae, Cyperaceae, Rosaceae, Amaryllidaceae, and Fabaceae (comprised > 81% of plant above‐ground biomass), with the remaining species classified as forbs (Appendix S1). Subsequently, samples from the 10 quadrats in each plot were combined. We calculated Shannon–Wiener Diversity Index (*H*′) to represent species diversity. The calculation method is as follows (Magurran 1988):

(1) $$p_{i}=n_{i}/N$$

(2) $$H^{'}=-\sum_{i=1}^{S}p_{i}\ln p_{i}$$

Where $p_{i}$ refers to the relative abundance of the *i* ^th^ species in the community, $n_{i}$ refers to the number of individuals of the *i* ^th^ species, *N* refers to the total abundance of the community, *S* refers to the species richness of the community, and higher values of *H*′ indicate greater species diversity.

Vegetation samples were dried in an oven at 60°C for 48 h and then ground to pass a 1‐mm screen for subsequent forage quality determination (Krizsan and Huhtanen 2013). Crude protein (CP) was determined using the Kjeldahl digestion method (Kjeltec 2300, Hoganas, Sweden), while neutral detergent fiber (NDF) and acid detergent fiber (ADF) were measured following Van Soest's method (Soest, Robertson, and Lewis 1991), incorporating heat‐stable alpha‐amylase and sodium sulfite in the NDF procedure.

### 2.4 Animal Daily Grazing Time

We used accelerometry data to assess activity patterns. A total of 24 animals with similar body weights were selected from the three strategies, with 6 animals from LA and EA, and 12 animals from LM/EM. All animals were equipped with tri‐axis accelerometers (Chengdu Druid Technology Co. Ltd.).

The resulting sum of all three axes (X: surge, Y: sway, Z: heave) yielded a value for the overall dynamic body acceleration (ODBA) as following:

(3) $$\begin{array}{cc} \text{ODBA}= & |\text{Dynamic acceleration}\;\left(x\right)|+|\text{Dynamic acceleration}\;\left(y\right)|\\ & +|\text{Dynamic acceleration}\;\left(z\right)| \end{array}$$

To better distinguish the behaviors of the animals, we conducted three observations each month, with each session lasting about 13 h (from 0530 h to 1830 h). We recorded any changes in behavior and the time they occurred. Using ODBA as a classification criterion, we accurately classified grazing behavior (> 0.1 g) and non‐grazing behavior (< 0.1 g). Based on this classification system, we calculated the daily grazing time for each animal (Chen et al. 2021; Yu and Klaassen 2021).

### 2.5 Feed Intake and Organic Matter Digestibility

A total of 36 animals (18 individuals from LM/EM and nine individuals from both EA and LA) were used to determine feed intake and organic matter digestibility of feed (including animals subjected to activity pattern observations). The determination of fecal output was conducted using an external marker TiO_2_, which is assumed to be inert in the digestive tract and evenly distributed in digesta. It is commonly employed in digestibility studies to avoid total feces collection or to measure the passage of digesta (Sankhyan et al. 1999). The marker was orally administered to animals as a daily dose of 2.5 g in a capsule during the three phases of 5–15 July, 5–15 August, and 5–15 September. Daily fecal grab samples, from the last 5 days of each phase, were pooled by animal and period (*n* = 108; 36 samples × 3 times). For each composite fecal sample, determinations included the concentration of the external marker TiO_2_ (Glindemann et al. 2009; Müller et al. 2011), CP, and ash. Each fecal sample was dried for 48 h in an oven at 60°C, ground to pass a 1‐mm screen, and analyzed for TiO_2_ concentration (Storeheier, Mathiesen, and Sundset 2003); CP was determined as described above (see 2.3). Fecal organic matter (FOM) content was calculated by measuring the difference between the residue (ash) of the dry sample after overnight incineration at 550°C in a Muffle furnace and the dry sample (Soest, Robertson, and Lewis 1991).

The fecal output was calculated by the equation:

(4) $$\mathrm{FO}=2.5/T_{i}O_{2}$$

Total fecal output (FO; g·sheep^−1^·day^−1^) was calculated by dividing the daily dose of TiO_2_ indicator per animal (2.5 g) by the concentration of TiO_2_ in the feces (g·day^−1^).

Digestibility of ingested organic matter (OMD; %) was calculated from fecal CP concentration (FCP) using the regression equation of Wang et al. (2009):

(5) $$\mathrm{OMD}=\left[0.899-0.644*\exp\;\left(-0.5774*\mathrm{FCP}/100\right)\right]*100\%$$

(6) $$\mathrm{OMI}=\mathrm{FOM}/\left(1-\mathrm{OMD}\right)$$

where organic matter intake (OMI; g·sheep^−1^·day^−1^) was then estimated by dividing FOM (OM basis) by 1‐OMD.

### 2.6 Diet DNA Metabarcoding Sequence Analysis

We quantified animal diet composition using fecal DNA metabarcoding. Additional fecal samples were collected and placed into 1.5 mL tubes in liquid nitrogen containers during the measurement of feed intake and kept in a −80°C freezer for further processing (*n* = 108, 36 samples × 3 times). A 0.2 g sample of feces was used for DNA extraction with QIAamp Fast DNA Stool Mini Kit (50, QIAgen GmbH), and an extraction blank was processed to monitor for cross‐contamination. DNA was quantified using the NanoDrop‐2000 UV–Vis Spectro‐photometer (Thermo Scientific, Wilmingtoo, DE, USA).

The chloroplast *rbcL* gene was used for DNA metabarcoding sequencing, with primers *rbcL* P1‐F (CTTACCAGYCTTGATCGTTACAAAGG) and *rbcL* P1‐R (GTAAAATCAAGTCCACCRCG) (Erickson et al. 2017; Omonhinmin and Onuselogu 2022; Trujillo‐Argueta, del Castillo, and Velasco‐Murguía 2022). For the polymerase chain reaction (PCR) assays, 10 μL reaction mixtures were prepared for each sample, including 0.3 μL of each primer, 0.2 μL KOD FX Neo polymerase, 2 μL dNTPs, 5 μL KOD FX Neo buffer, and 50 ng of DNA template. The thermal cycling program consisted of initial denaturation at 95°C for 5 min, followed by 40 cycles of denaturation at 94°C for 20 s, annealing at 55°C for 30 s, extension at 72°C for 1 min, and a final extension at 72°C for 5 min. All PCRs included a no‐template negative control. Each primer was labeled with a 16‐nucleotide multiplex identifier tag at the 5′ end, differing from the other tag by 8 nucleotides, allowing for unique labeling of PCR products. Sequencing was performed on the Illumina HiSeq 2500 platform.

Sequence quality control and preliminary identification were conducted by Trimmomatic v0.33 (Usadel Lab, GE), where sequences with an average Illumina fastq quality score < 20 were not considered. Cutadapt software (TU Dortmund University, DE, and National Bioinformatics Infrastructure Sweden, SE) was used to identify and remove primer sequences, with parameters allowing a maximum mismatch rate of 20% and a minimum coverage of 80% for primer sequence identification. The processed data were denoised using the DADA2 method in QIIME2 (Bolyen et al. 2019; version 2020.6). Using the filterAndTrim function for further quality control of the data, maxEE is set to 2 {where EE = sum [10^(−*Q*/10)]}. The learnErrors function is used to build the model, the dada function for denoising, and the mergePairs function for merging paired‐end reads. Identical sequences were collapsed, and plant species were assigned to reference sequences based on their unique matches (100% identity) with DNA metabarcoding sequences. Only unique sequences with 100% identity to reference sequences were retained for further analysis. The most refined taxonomic assignments were made using the Naive Bayes classifier with the Fungene and NCBI databases. The summarize_taxa command was used to group identical sequences, perform within‐sample statistics, and quantify the relative read abundance of each sequence. The proportion of each amplicon sequence variant (ASV) in each sample represents the relative abundance of the components of the animal's diet (DRA). This method is widely used for quantifying proportions of consumed foods in animals feeding studies and has been validated in multiple studies (Guo et al. 2021; Spitzer et al. 2023; Walker et al. 2023).

We converted botanical dietary compositions to nutritional compositions by multiplying the proportion of each plant family in a fecal sample by its nutritional content (follow the methods of Felton et al. 2016; Spitzer et al. 2023). We calculated dietary richness (number of plant species consumed) through bootstrap iterations, representing the average number of ASVs present in each animal's standardized dietary sample (Walker et al. 2023).

### 2.7 Measurement of Food Availability and Selectivity

The proportion of each plant family to total above‐ground biomass was quantified, representing the relative abundance of vegetation components (VRA), also providing a quantitative measure of food availability. This data could then be compared to the proportions of food items in the animal's diet. To assess the selectivity of the animal, we applied Jacobs' index (*D*) (Jacobs 1974), in which the utilization of a food item (its proportion in the diet, *r*) is related to its relative availability in the vegetation (*p*) according to the following equation:

(7) $$D=\left(r-p\right)/\left(r+p-2\mathrm{rp}\;\right)$$

The index ranges from −1 to 1, with negative values indicating utilization below relative availability (“avoidance”) and positive values indicating utilization above relative availability (“preference”); a value of 0 corresponds to the utilization of a food item in proportion to its relative availability.

### 2.8 Statistical Analysis

We used t‐tests to assess differences in feed intake, organic matter digestibility, and daily grazing time between LA and LM strategies as well as between EA and EM strategies. Additionally, we examined the differences between diet nutrient (NDF, ADF, and CP) and vegetation nutrient contents. Data for each grazing strategy were obtained by averaging the measurements from three sheep per plot (with three replicates per plot). Sampling was conducted three times, resulting in nine values per group. In addition, the differences in vegetation characteristics under different grazing strategies were determined using one‐way analysis of variance (ANOVA). To account for the non‐normal distribution of selectivity data, we conducted Wilcoxon tests to evaluate whether Jacobs' *D* for forages differed significantly from zero. These statistical analyses were conducted using SPSS 22.0 (IBM Corp., Armonk, NY, USA).

We calculated Bray–Curtis dissimilarities and ordinated the results using nonmetric multidimensional scaling (NMDS) between LA and LM, LM and EM, as well as between EA and EM strategies. Subsequently, we employed perMANOVA (permutational multivariate ANOVA) from the vegan R package to test for dietary differences. Four multiple regression models (LA, LM, EA, and EM) were established to determine the relationship between vegetation characteristics and dietary richness. Significance of the explanatory variables was assessed using Wald statistics. All multivariate analyses were conducted in R version 3.6.1 (R Core Team 2019).

## 3 Results

### 3.1 Animal Feeding Behavior

**FIGURE 2:**
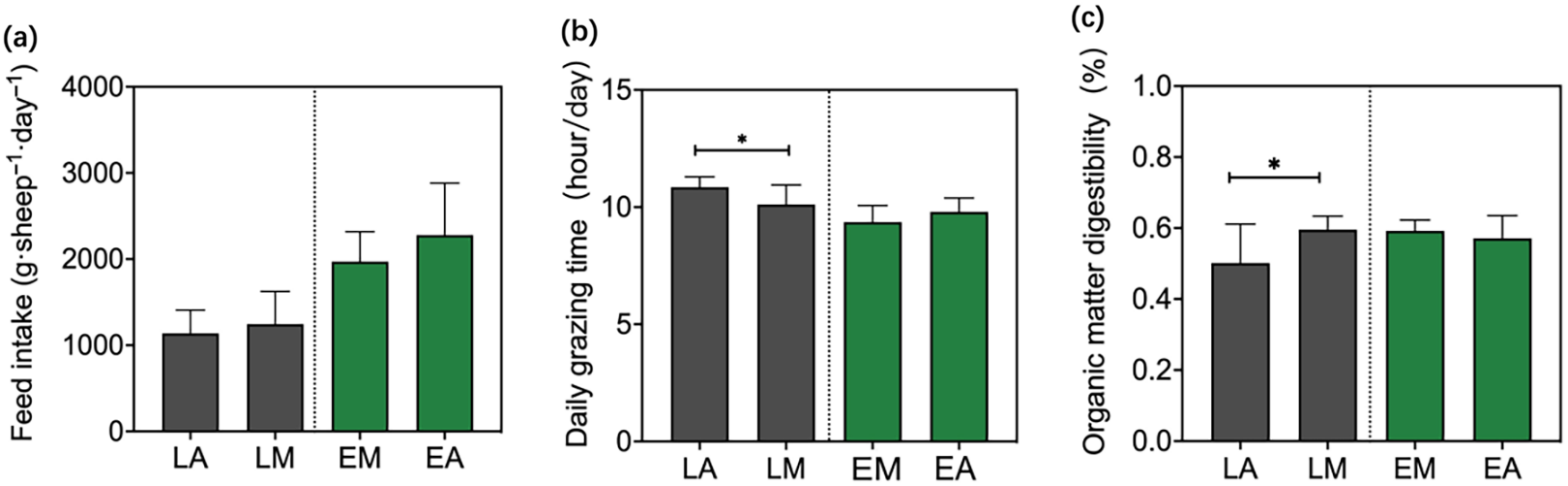
Feeding behavior as determined by feed intake (a), daily grazing time (b), and organic matter digestibility (c) of animals within different grazing strategies (EA = ewes alone; EM = ewes in mixed‐grazing; LA = lambs alone; LM = lambs in mixed‐grazing). Values represent mean ± SE, and asterisks indicate significant differences at 0.05 (*), 0.01 (**), and 0.001 (***).

From the comparison of feed intake (Figure 2a), daily grazing time (Figure 2b), and organic matter digestibility (Figure 2c), there was not a significant difference in feed intake between LA and LM, but the organic matter digestibility in LM was significantly higher than that in LA (*t* = 2.425, df = 16, *p* = 0.036), while their daily grazing time was significantly lower than that in LA (*t* = −2.357, df = 16, *p* = 0.031). There were not significant differences in feed intake, organic matter digestibility, and daily grazing time between EA and EM.

### 3.2 Feeding Selection and Nutritional Intake

**FIGURE 3:**
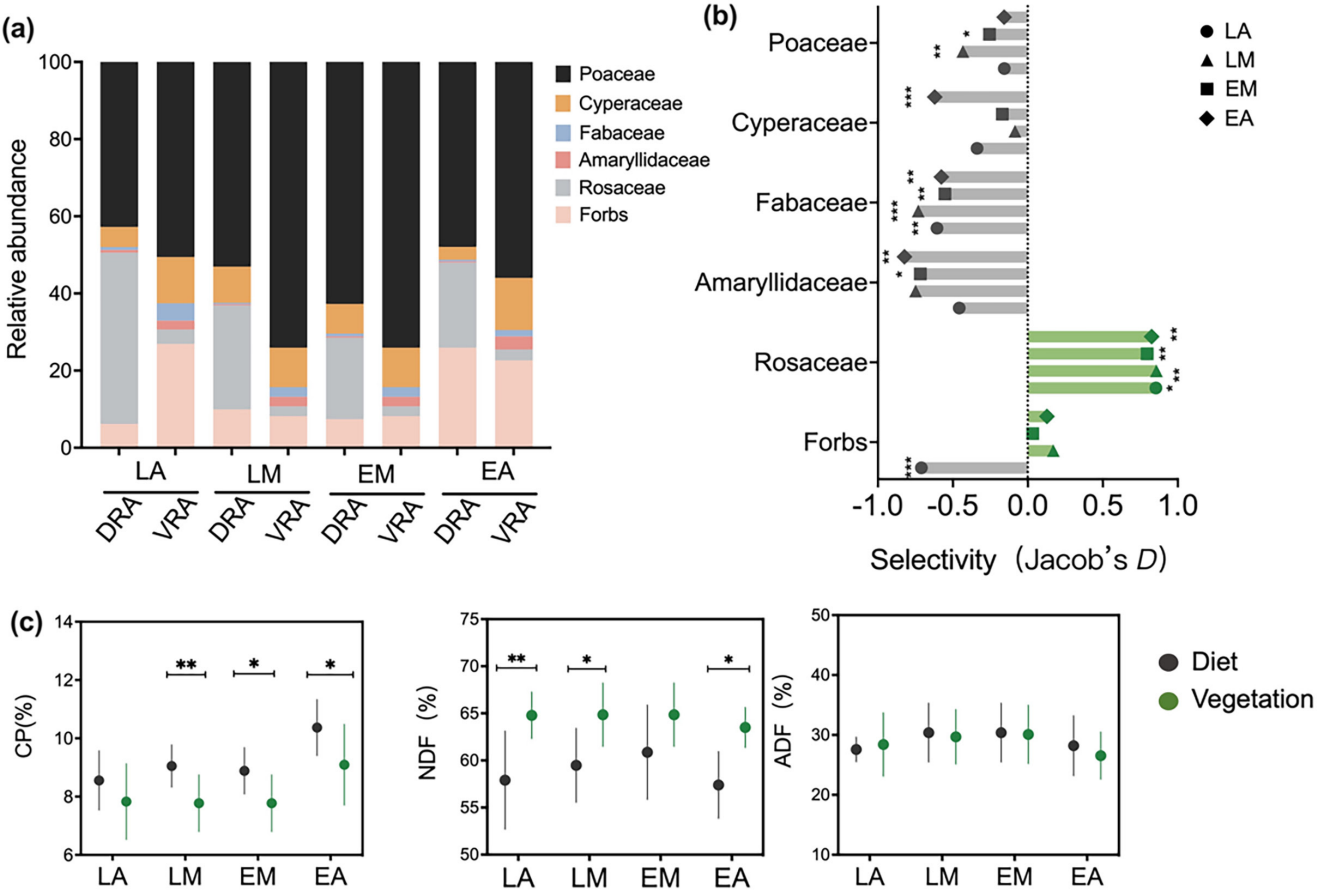
Mean relative abundance of plant families and forbs for animal diets (DRA) and vegetation (VRA) within the three grazing strategies (designations follow Figure 2) (a). Mean animal food selectivity for plant families and forbs based on Jacob's *D* index, where negative values indicate diet proportions lower than availability (“avoidance,” in gray) and vice versa (“preference,” in green), and zero values indicate when food items are the same proportion in their diets and the vegetation (b). Comparison of mean (±SE) crude protein (CP), neutral detergent fiber (NDF), and acid detergent fiber (ADF) for diets and vegetation within grazing strategies (c). Asterisks indicate significant differences at *α* = 0.05 (*), 0.01 (**), and 0.001 (***).

The dietary composition of LM and EM was similar, with Poaceae accounting for over 50%, and the remaining components including Cyperaceae, forbs, and Rosaceae. For LA, diets were almost entirely dominated by Poaceae (approximately 43%) and Rosaceae (approximately 44%), while dietary composition of EA was primarily characterized by Poaceae (48%) and forbs (26%; Figure 3a). We also observed that LM, EM, and EA exhibited a preference for forbs (Jacobs' *D* > 0), while only LA significantly “avoided” forbs intake (Jacobs' *D* < 0; Figure 3b). NDF content was significantly lower than the levels provided by the vegetation in the diets of LA, LM, and EA (*t* = −3.381, df = 16, *p* = 0.004; *t* = −2.937, df = 16, *p* = 0.010; and *t* = −4.179, df = 16, *p* = 0.001; respectively), while ADF content in diets was generally consistent with those in vegetation (Figure 3c). Significant differences in CP between diets and vegetation were observed for LM, EM, and EA (*t* = 2.974, df = 16, *p* = 0.009; *t* = 2.499, df = 16, *p* = 0.024; and *t* = 2.4771, df = 16, *p* = 0.014; respectively), but not for LA.

### 3.3 Dietary Dissimilarity Between Animals

**FIGURE 4:**
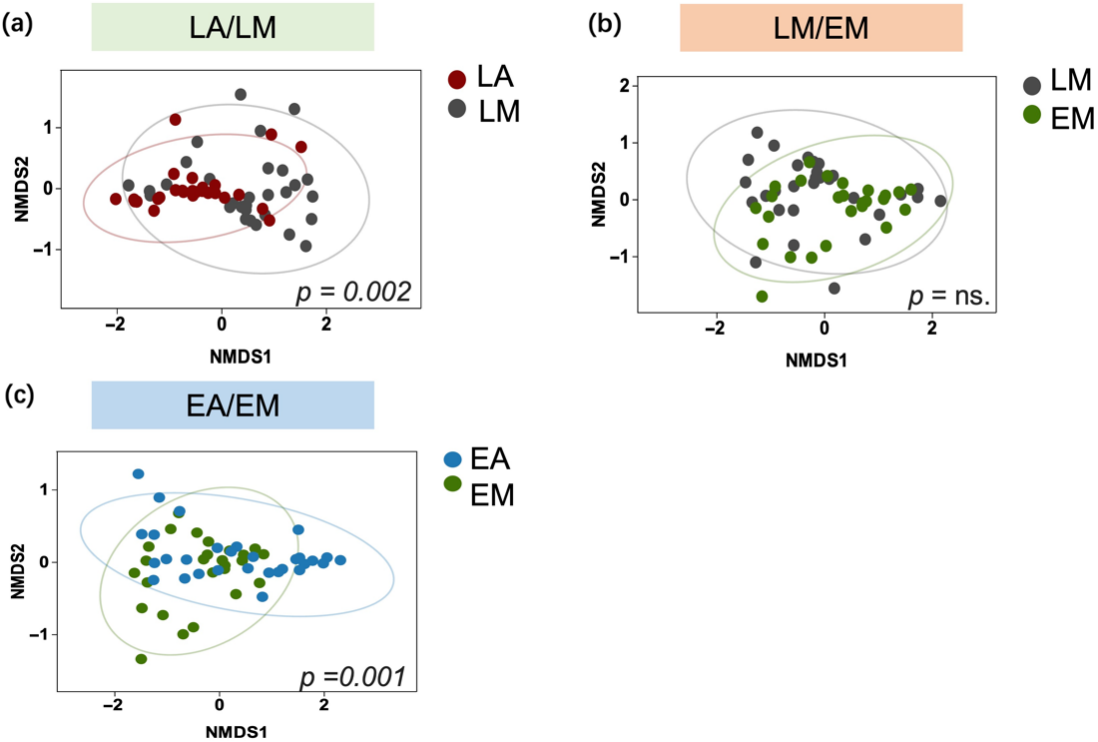
Dietary dissimilarity among grazing strategies (designations follow Figure 2) illustrated by nonmetric multidimensional scaling (NMDS) ordination. Solid circles represent individual dietary samples with different colors distinguishing lambs diets (a), lambs versus ewe diets (b), and ewe diets (c). Closer sample positions within the ordination indicate more similar diets. Elliptical boundaries represent the 95% confidence interval for diets, and *p*‐values indicate results of perMANOVA analyses.

NMDS and perMANOVA showed significant differences between LA and LM (stress = 0.072; perMANOVA, *R* ^*2*^ = 0.125, *p* = 0.002; Figure 4a) as well as between EA and EM (stress = 0.080; perMANOVA, *R* ^*2*^ = 0.169, *p* = 0.001; Figure 4c). There were not significant dietary differences between lambs and ewes in the LM/EM strategy (Figure 4b).

### 3.4 Relationship Between Vegetation Characteristics and Dietary Richness

**FIGURE 5:**
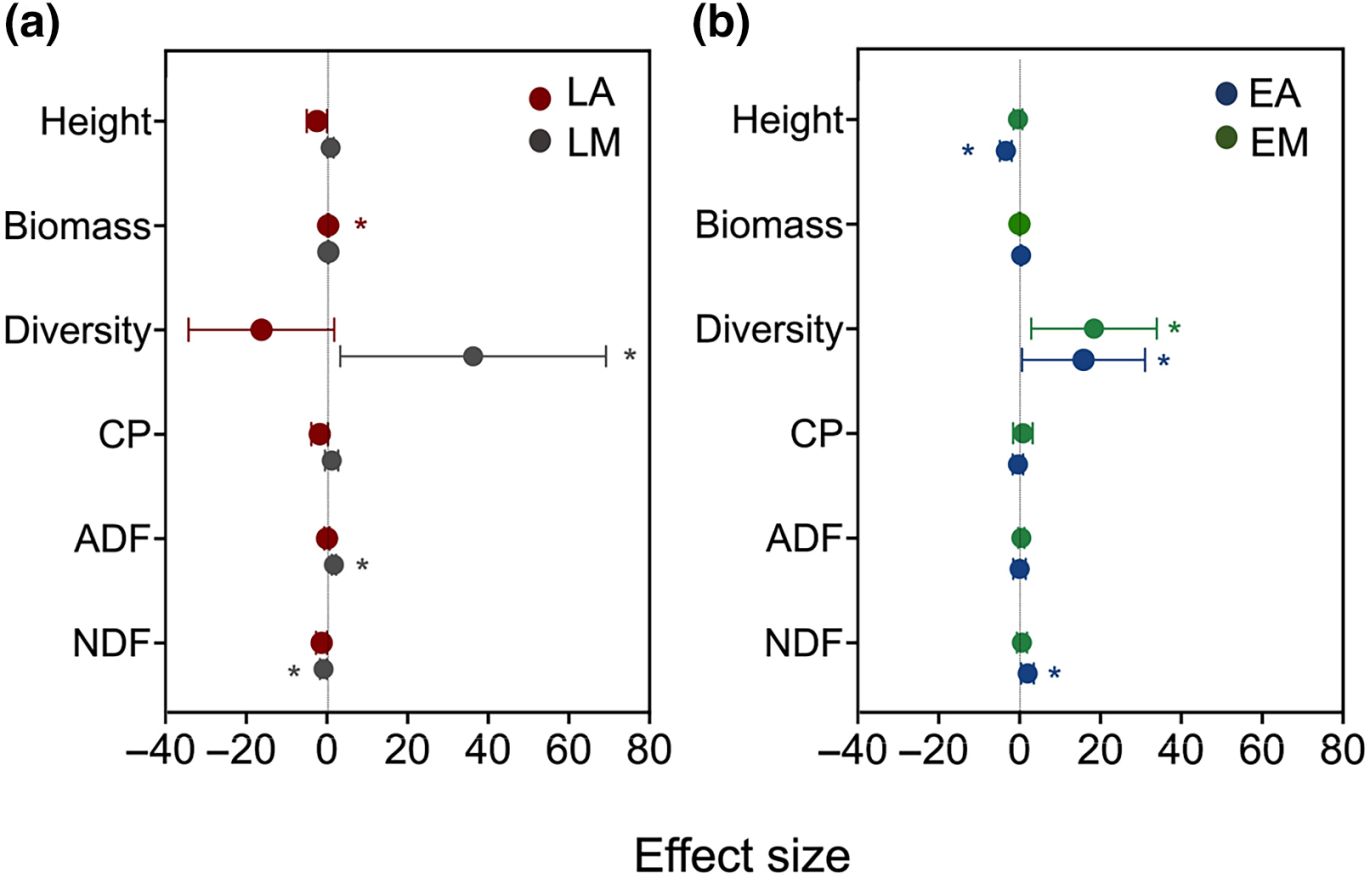
Results of multiple linear regression models assessing the effects of vegetation characteristics (height, biomass, and diversity) and forage nutritional factors [crude protein (CP), neutral detergent fiber (NDF), and acid detergent fiber (ADF)] on dietary richness for lamb (a) and ewe grazing (b) (designations follow Figure 2). The effect sizes are regression coefficients based on the linear models. The adjusted *R*‐squared values are 0.569 in the LM group and 0.447 in the LA group. For the ewe group, the adjusted *R*‐squared value is 0.673 in the EM group and 0.714 in the EA group. These values suggest varying degrees of model fit across the different group and model configurations. Symbols indicate the magnitude and direction of the effect on dietary richness on each estimate, while error bars represent 95% confidence intervals around these estimates. Asterisks indicate significant differences at *α* = 0.05 (*).

Apart from the higher species diversity in the LA grazing grassland compared to other grazing strategies (*p* = 0.007), there were no significant differences in above‐ground biomass, species height, community CP, NDF, and ADF content (Appendix S2). The dietary richness of LM was significantly correlated with species diversity (*p* = 0.042), NDF (*p* = 0.014), and ADF (*p* = 0.001), with species diversity having the strongest effect among these indicators. In contrast, the dietary richness of LA was only influenced by above‐ground biomass (*p* = 0.048), and species diversity showed a negative but non‐significant effect (Figure 5a). Additionally, the dietary richness of EA was significantly and negatively correlated with vegetation height (*p* = 0.001) and significantly positively correlated with vegetation NDF content (*p* = 0.041) and species diversity (*p* = 0.031). For EM, dietary richness was only influenced by vegetation species diversity (*p* = 0.043). In both the EA and EM strategies, species diversity had the strongest effect on dietary richness.

## 4 Discussion

Quantitative analysis of dietary preferences identified differences in the dietary composition among the grazing strategies. In particular, the mixed‐grazing strategy influenced lamb dietary selection, further impacting their nutrient intake. When lambs grazed with ewes, they likely learned from experienced ewes to efficiently forage and achieve high digestibility by selectively consuming less abundant, but nutrient‐rich forbs within the community.

### 4.1 Species Selection Among Different Grazing Strategies

In most grazing ecosystems, managers often group livestock based on gender and age. However, this management approach often ignores the low resource utilization capacity and immature feeding strategies of young animals (Miranda‐de La Lama, Villarroel, and María 2012). Consistent with research by Bolzan et al. (2020), we found that combining young and adult animals can strongly influence the forage preferences of young animals (Figure 4). Lambs in mixed‐grazing mimicked the foraging preference of ewes, consuming a higher proportion of forbs than what vegetation provided, while exhibiting an “avoidance” of dominant grasses in the vegetation. However, this phenomenon was not observed in LA (Figure 3b) and agrees with a previous study that reported inexperienced lambs were less likely to learn to mix high‐nutrient foods together (Villalba, Provenza, and Han 2004). On the contrary, animals with rich experience consume all plants in an area, and they are more likely to select a greater variety of species combinations (Provenza et al. 2003). Because maternal care has an important impact on the foraging behavior and nutritional ecology of herbivores when grazing in mixed groups, and has an important impact on the growth and development of herbivores (Berry et al. 2019). These results indicate that different management systems can alter the foraging behavior of animals.

### 4.2 Impact of Vegetation Characteristics on Animal Feeding Selection

As vegetation diversity increases, grazing animals have a greater chance of selecting preferred food and to forage for a diverse diet, which positively affects their consumption of food and enhances nutrient intake by modifying nutrient balance (Feng et al. 2016; Wang et al. 2008). However, we found that the dietary richness of ewes was influenced by vegetation species diversity, while the dietary richness of lambs exhibited differences between the mixed‐grazing and lambs‐alone strategy. The dietary richness of lambs in the mixed‐grazing was similar with ewes, but the dietary richness of LA was only affected by vegetation above‐ground biomass, even though the species diversity in their grazing area is higher (Figure 5; Appendix S2). Some studies suggest that inexperienced lambs do not consume plant species based on the overall nutrition of grassland, leading animals to consume few plant species and reducing grazing extent, which leads to non‐uniform utilization of grasslands (Villalba, Provenza, and Han 2004). Consistent with the findings of Mohammed et al., our study revealed that differences in animal grazing time are jointly influenced by forage quality in the grazing area and selective foraging behavior (Mohammed et al. 2020). Lambs in mixed‐grazing with adult ewes benefit from consuming a wider variety of plant species rather than spending more time grazing (Berry et al. 2019). However, for young animals, high species diversity can confuse their discrimination, increase foraging costs, and reduce their ability to select optimal food (Provenza et al. 2003). From this perspective, high vegetation species diversity cannot be effectively utilized by lambs when grazing alone in complex environments. In contrast, experienced adult ewes influence lambs within a mixed‐grazing strategy to consume diets with higher digestibility using less feeding time (Figure 2).

### 4.3 Selective Forage Leads to Differences in Nutrient Intake

Diet selection primarily occurs due to an animals' effort to meet their nutritional requirement (Westoby 1978). Therefore, large herbivores obtain high quality food from heterogeneous vegetation through selective consumption (Jamieson and Hodgson 1979). When animals can select a diverse array of plants for their diets, they are more likely to reach their nutritional needs and consume plants with higher quality (Spitzer et al. 2020). Previous studies have shown that large herbivores such as moose, yaks, horse, and sheep increase their nutritional intake by selectively feeding on higher nutrient content (Guo et al. 2021; Spitzer et al. 2023; Walker et al. 2023;). We found that CP intake by ewes and lambs in the mixed‐grazing strategy was higher than that provided by vegetation, while NDF intake was lower (Figure 3c). Clearly, this pattern was not observed in lambs‐alone. González et al. found that adult ewes exhibited significantly higher selectivity for plants with low fiber content and high digestibility compared to lambs. The differences in forage selection between lambs and adult ewes were significantly associated with their body size differences and foraging experience (González‐Pech et al. 2021). This indicates that lambs grazing together with ewes impact both the plant species selection and the nutrient intake.

This study suggests that a mixed‐grazing strategy allows animals to achieve higher nutritional intake. This approach not only promotes animal welfare but also enhances the comprehensive utilization of the plants available on the pasture. Enhancing grassland species diversity and maintaining well‐maintained natural pastures are essential prerequisites for improving animal production efficiency and increasing herders' income. However, our study has certain limitations. It was conducted in a temperate grassland region, focused solely on one sheep breed, and spanned only 1 year. Thus, further exploration would be valuable to assess whether these findings are applicable to multi‐species, long‐term studies and whether they can be generalized to other ecosystems.

## 5 Conclusions

We found that LA significantly avoid selecting forbs with low fiber content and high CP content in the vegetation, which increased their daily grazing time and consumption of grass with low digestibility. Interestingly, in mixed‐grazing, lambs showed a preference to consume high‐nutrient forage from a diverse species of forbs, which increased their adaptability to vegetation heterogeneity in grasslands. This study provides new insights into the relationship between grassland ecosystems and the feeding behaviors of animals. Furthermore, the management practice of lambs and ewes grazing together can be used to improve forage strategies for lambs and enhance their ability to obtain food resources in native grassland systems.

## Author Contributions

**Pengzhen Li:** formal analysis (lead), writing – original draft (lead). **Zhenhao Zhang:** methodology (supporting). **Thomas A. Monaco:** visualization (supporting), writing – review and editing (supporting). **Yuping Rong:** funding acquisition (lead), supervision (lead), visualization (lead).

## Conflicts of Interest

The authors declare no conflicts of interest.

## Supporting information


Data S1.

Data S2.

## Data Availability

The main data has been uploaded as Data S1 and S2 and will be freely available upon acceptance.
