# Supplementary material for: Lambs Grazing With Adult Ewes Prefer Forbs With High‐Nutrient Content in Native Grasslands Dominated by Leymus chinensis and Stipa grandis
Source: Ecol Evol. 2024 Nov 18;14(11):e70609. doi: 10.1002/ece3.70609 (PMC11573460; doi:10.1002/ece3.70609)
Supplement: Supplementary file 1 — Data S1. [file ECE3-14-e70609-s002.docx]

**Appendix S1.** Plant species composition and mean (± SE) above-ground biomass for three grazing strategies (LA: Lambs alone, LM/EM: mixed lambs and ewes, EA: ewes alone).

| **Plant Species** | **Family** | **Plant**  **Functional Group** | **Above-ground Biomass(g/m^2^)** | | |
| --- | --- | --- | --- | --- | --- |
|  |  |  | **LA** | **LM/EM** | **EA** |
| *Stipa grandis* | Poaceae | Grass | 7.34±3.45 | 8.81±2.45 | 24.17±6.84 |
| *Leymus chinensis* |  | Grass | 10.39±3.75 | 20.05±5.72 | 8.74±4.33 |
| *Cleistogenes squarrosa* |  | Grass | 4.46±1.24 | 0.81±0.58 | 2.91±1.53 |
| *Agropyron cristatum* |  | Grass | 1.90±0.97 | 0.34±0.09 | 0.87±0.26 |
| *Achnatherum sibiricum* |  | Grass | 0.21±0.07 | - | 0.16±0.09 |
| *Koeleria macrantha* |  | Grass | 0.26±0.12 | - | - |
| *Carex korshinskyi* | Cyperaceae | Sedge | 6.30±1.39 | 7.83±0.92 | 5.45±1.91 |
| *Melilotus officinalis* | Fabaceae | Legume | 1.32±0.29 | 0.45±0.20 | 0.76±0.12 |
| *Astragalus galactites* |  | Legume | 0.13±0.14 | 0.01±0.07 | 0.14±0.04 |
| *Thermopsis lanceolata* |  | Legume | 0.21±0.12 | 0.18±0.11 | 0.06±0.00 |
| *Astragalus adsurgens* |  | Legume | - | - | 0.14±0.08 |
| *Caragana microphylla* |  | Legume | 0.50±0.04 | - | - |
| *Gueldenstaedtia verna* |  | Legume | 0.08±0.05 | 0.07±0.04 | 0.01±0.00 |
| *Astragalus complanatus* |  | Legume | 0.01±0.00 | - | 0.01±0.01 |
| *Allium ramosum* | Amaryllidaceae | Forb | 0.97±0.11 | 1.13±0.05 | 0.55±0.17 |
| *Sibbaldianthe bifurca* | Rosaceae | Forb | 1.29±0.20 | 1.06±0.04 | 0.78±0.04 |
| *Sibbaldia procumbens* |  | Shrub | - | 0.08±0.01 | - |
| *Artemisia annua* | Asteraceae | Forb | 2.23±0.03 | 2.01±0.00 | 2.63±0.01 |
| *Aster altaicus* |  | Forb | 2.02±0.01 | 1.03±0.02 | 0.08±0.02 |
| *Saussurea japonica* |  | Forb | - | 0.71±0.01 | - |
| *Artemisia scoparia* |  | Forb | 2.10±0.02 | 0.82±0.01 | - |
| *Bupleurum chinense* | Apiaceae | Forb | 1.25±0.14 | - | - |
| *Androsace umbellata* | Primulaceae | Forb | 1.16±0.08 | - | - |
| *Convolvulus ammanii* | Convolvulaceae | Forb | 1.16±0.07 | 0.49±0.08 | 0.28±0.14 |
| *Atriplex dimorphostegia* | Amarantaceae | Forb | - | 0.65±0.00 | - |
| *Salsola collina* |  | Forb | 0.84±0.07 | 0.84±0.02 | 0.02±0.01 |
| *Kochia scoparia* |  | Forb | 0.77±0.04 | - | 0.04±0.02 |
| *Chenopodium album* |  | Forb | 0.42±0.00 | 0.74±0.02 | 0.04±0.02 |
| *Iris lactea* | Iridaceae | Forb | 0.06±0.03 | 1.80±0.06 | 0.06±0.02 |
| *Iris tenuifolia* |  | Forb | 0.04±0.00 | 0.08±0.00 | 0.01±0.00 |
| *Rumex acetosa* | Polygonaceae | Forb | - | 1.27±0.15 | - |
| *Polygonum divaricatum* |  | Forb | - | 0.15±0.09 | - |
| *Tribulus terrestris* | Zygophyllaceae | Forb | - | 0.01±0.01 | 0.14±0.08 |
| *Cymbaria dahurica* | Scrophulariaceae | Forb | 1.12±0.75 | - | 0.21±0.12 |
| *Eremurus chinensis* | Brassicaceae | Forb | - | - | 0.01±0.00 |
| *Lepidium latifolium* |  | Forb | - | 1.58±0.02 | 0.07±0.06 |
| *Ruta graveolens* | Rutaceae | Forb | 0.01±0.03 | - | - |

**Appendix S2.** Vegetation characteristics (above-ground biomass (g/m^2^), species diversity, height (cm), CP, NDF and ADF content(%); mean values ± SE) of community under different grazing strategies (LA: Lambs alone, LM/EM: mixed lambs and ewes, EA: ewes alone).

| **Characteristics** | **LA** | **LM/EM** | **EA** |
| --- | --- | --- | --- |
| Above-ground biomass | 48.52±7.61 | 53.02 ±11.83 | 48.21 ±14.62 |
| Species diversity | 0.99^a^±0.26 | 0.67^b^ ±0.09 | 0.70^b^ ±0.20 |
| Height | 12.52 ±2.67 | 11.08 ±3.84 | 9.54 ±1.73 |
| CP | 8.02 ±2.16 | 8.04 ±1.93 | 9.08 ±2.33 |
| NDF | 64.79±2.47 | 64.28 ±3.09 | 63.78±1.98 |
| ADF | 28.40±5.29 | 29.99 ±5.02 | 28.72 ±4.62 |

Different letters indicate significantly differences (*p*<0.05).
